# Edible Plant-Derived Exosome-like Nanoparticles as Prebiotic Nanocarriers: Gut Microbiota Modulation and Functional Food Potential

**DOI:** 10.3390/pharmaceutics18050520

**Published:** 2026-04-24

**Authors:** Yağız Alkan, Yalçın Mert Yalçıntaş, Mikhael Bechelany, Sercan Karav

**Affiliations:** 1 Department of Molecular Biology and Genetics, Çanakkale Onsekiz Mart University, Çanakkale 17000, Turkey; ygz.lkn.1@gmail.com (Y.A.); yalcinmertyalcintas@stu.comu.edu.tr (Y.M.Y.); 2 European Institute for Membranes (IEM), Unité Mixte de Recherche-5635, University of Montpellier, École Nationale Supérieure de Chimie de Montpellier, Centre National de la Recherche Scientifique, Place Eugène Bataillon, CEDEX 5, F-34095 Montpellier, France

**Keywords:** exosome, plant-based, prebiotic, gut microbiome, nanomaterial

## Abstract

The gut microbiota takes charge in a pivotal role in metabolic equilibrium, immune response, and modulating gut lining stability and has become the main focus of nutrition and functional food research. In this regard, the definition of prebiotics has progressed past the traditional approach limited to indigestible dietary fibers, embracing more targeted, biologically active, and functional delivery systems. In recent years, plant-derived exosomes (PDEs), a subclass of exosomes defined as extracellular vesicles (EVs) in the 30–150 nm size range, have emerged as an innovative class of nanostructures supporting this transformation. Plant-derived exosome-like nanoparticles (PELNs) have been taken into account as natural nanocarriers which are suitable for the gastrointestinal system with the help of their high biocompatibility, low immunogenicity profiles and rich bioactive cargo contents. This review discusses structural features of PELNs, molecular cargo content, and biological roles comprehensively and focuses especially on gut microbiota interactions. MicroRNAs, proteins, lipids, polyphenols, and glycans which PELNs contain are discussed with regard to shaping the microbial composition, regulating microbial metabolic activity, and modulating host-microbe communication. Findings derived from in vitro, in vivo, and limited translational studies indicate that PELNs can modulate specific microbial taxa, increase short-chain fatty acid (SCFA) yield, strengthen mucosal immune homeostasis, and induce source-dependent responses in the gut microbiota. In their traditional definition, prebiotics are taken into account as food components which selectively support proliferation and metabolism of helpful microbes, especially Bifidobacteria and Lactobacilli. Within this framework, PELNs are not only passive carriers of functional components but also evaluated as active systems which can directly affect microbiota composition and metabolic functions. Thus, they are repositioned as “prebiotic nanocarriers.” Also this review evaluates the potential of functional food and integration of major edible PELNs into synbiotic formulations by discussing their isolation and characterization methods and stabilities in the gastrointestinal environment. Limitations of clinical applications and lack of research from a prebiotic nanocarrier perspective of PELNs show that this field still contains important research gaps. The novelty of the study lies in its integration of PELN research with nutrition-based approaches to microbiota modulation and innovative functional food strategies under a single multidisciplinary conceptual framework.

## 1. Introduction

The gut microbiota is an extremely dynamic and complex microbial ecosystem consisting of bacteria, archaea, fungi, viruses, and some eukaryotes that exist in a symbiotic relationship with the host organism [1,2,3,4,5]. This community, which begins to take shape at birth, follows a distinct succession process, especially during the first three years of life [4,6]. In babies born vaginally, facultative anaerobic bacteria colonize in the early stages, while *Bifidobacterium* species become dominant within the first six months. A rich diversity of species from the main human intestinal phyla develops when solid foods are introduced into the diet [6,7]. A full-fledged microbiome helps break down dietary fiber and other carbohydrates that are not able to be digested by host enzymes, optimizes food absorption, enhances the immune system, regulates metabolic processes, and prevents the colonization of harmful organisms [4,6,8]. The function and profile of the gut microbiota can be modulated by diverse factors. Those factors could be natural or human, such as method of birth, feeding practice, gestational age, maternal microbiome and nutrition, antibiotic use, environmental exposures, and lifestyle [4,6]. Dysbiosis is related to various chronic conditions. Asthma, atopic diseases, diabetes, obesity, inflammatory bowel diseases (IBD), cardiovascular diseases, and neuropsychiatric disorders can be cited as examples [8,9]. Dysbiosis can lead to many severe problems related to intestinal composition, the immune system, and metabolic homeostasis.

The composition of microbiota has a diverse biological effect that goes beyond existing gastrointestinal functions [8]. It converts dietary components and endogenous substrates into functional metabolites like SCFAs (acetate, butyrate, and propionate), indoles, and bile acids. Thus epigenetic mechanisms, gene expression, immune responses, and disease pathogenesis get affected [8]. These metabolites can modulate some biochemical processes along the gut–brain axis, including neurotransmitter production, shape endocrine responses, and regulate physiological processes in distant organs [8]. Today, using metagenomic, metatranscriptomic, and metaproteomic approaches, millions of microbial genes have been identified. This reveals microbial signatures that vary between countries and individuals [4,7]. With age, variations in the profile of the microbiota are observed. An elevation in *Bacteroides* and certain *Clostridium* groups is reported among older individuals [7]. These changes have been found to be intimately related to health complications linked to age. In order to enhance healthy aging, the significance of protecting the structure and metabolic activity of the microbiota has been underscored.

The gut microbiota represents a pivotal element, governing a wide range of essential biological processes, from immune modulation to barrier integrity and from the management of systemic signaling networks to nutrient metabolism. Strategies that support the healthy development of this ecosystem may contribute to mitigating disease risks and enhancing overall health in the long term. In this context, a range of strategies may be applied, such as promoting breastfeeding, balanced nutrition, lifestyle adjustments, prebiotic and probiotic applications, and fecal microbiota transplantation.

Prebiotic research has evolved from the initial discovery of the health benefits of dietary fiber to contemporary, nuanced understanding of its intricate mechanisms and multifaceted health effects [10,11,12]. Initial studies focused on traditional prebiotics. In the following years, human milk oligosaccharides (HMO), galactooligosaccharides (GOS), and resistant starch have also been identified [10,11,13]. Fructooligosaccharides (FOS), found in bananas and onions, and inulin, found in garlic, are resistant to undergoing fermentation in the colon and decomposition in the initial gastrointestinal segment. On the other hand, milk-derived GOS and HMO stimulate the expansion of beneficial bacteria [10]. SCFAs, for instance, acetate, propionate, and butyrate, are produced via the fermentation of these compounds. These elements inhibit the translocation of pathogens, strengthen the epithelial barrier function, and maintain intestinal integrity [11].

Technological innovations have diversified prebiotics’ spectrum of usage significantly while optimizing their production workflows. Spray drying, coacervation, freeze-drying, and emulsion are the microencapsulation techniques used. These techniques protect prebiotics from heat, moisture, and oxidation and also ensure their safe delivery to target areas and enable controlled release. Thus, these methodologies have facilitated extending the shelf life of prebiotics [10,14]. These methods enhance the effectiveness and stability of synbiotic formulations when combined with probiotics [11,14]. The emergence of nanotechnology is one of the most remarkable developments in the research of prebiotics. Systems of nanoparticles and nanocapsules increase the stability, bioavailability, and solubility of prebiotics. Thus, they have made controlled and targeted release possible [10,14]. Thanks to nanotechnology, ‘smart carriers’ that combine prebiotics and probiotics can be developed. These carriers control the release of drugs at the precisely desired point during their journey through the gastrointestinal tract. As a result, the drug works more effectively at its target, while the body as a whole suffers less harm [14]. It is crucial for the nanomaterials to achieve “Generally Recognized as Safe” (GRAS) status, both in terms of commercialization and safety of health [14]. In summary, the evolution of prebiotics has been characterized by the integration of nanotechnology and biotechnology. In this process, exosomes play a pivotal role in the transfer of biomolecules and intercellular communication [15]. Plant-derived exosome-like nanoparticles (PELNs) suppress pathogens and support the proliferation of beneficial bacteria thanks to their bioactive components. Thus, they exhibit prebiotic activity. In this context, PELNs offer an innovative platform thanks to their properties, such as strengthening the strength of the intestinal seal, reducing permeability, suppressing inflammation, and improving microbiota balance.

PELNs are rising as a favorable option to overcome the limitations of mammalian-derived exosomes for drug delivery systems [16,17,18]. PELNs can be obtained from natural plants with short extraction cycles and high yields. Their low immunogenic potential and the absence of zoonotic pathogen transmission risk offer significant advantages [16,19]. They have a rich content of functional compounds like lipids, proteins, miRNAs, glycans, and polyphenols. This rich content of bioactive molecules reduces the probability of being detected as exogenous by the host immune system while also enhancing synergistic therapeutic effects [16,17,18]. Their double-layered lipid membrane structures preserve the integrity of the compounds they carry. Thus, this structure ensures stability even under harsh environmental and gastrointestinal conditions [16]. Their edible structure allows them to be safely introduced through different means, such as intravenous, intranasal, and oral. Whereby the risk of side effects or toxicity is reduced [16,19]. PELNs have greater biocompatibility and safety compared to synthetic nanocarriers. Also, they show lower cytotoxicity to healthy tissues. Owing to these characteristics, PELNs have become highly remarkable in both basic and clinical research but still require further validation [18]. They have been shown to be effective in transporting small molecules, drugs, or small RNA (sRNA) molecules; reducing inflammation; interspecies targeting; and regulating gene expression [19].

This review comprehensively investigates general characteristics and functional interactions related to gut microbiota of PELNs in the context of innovative nanocarrier systems. Both the ability to carry bioactive compounds and to direct microbiota composition directly have been emphasized. This review aims to make a unique and multidisciplinary contribution by integrating PELNs into nutrition-based strategies and functional foods.

In the literature, plant-derived vesicular structures have been described using various terms, including exosomes, PELNs, plant-derived extracellular vesicles (PEVs), and plant-derived nanovesicles (PDNVs), often depending on the isolation method and characterization approach. For clarity and consistency, the term “PELNs” is adopted throughout this review as a broader and more representative designation.

## 2. Structural and Molecular Composition of Edible Plant-Derived Exosome-like Nanoparticles

Extracellular vesicles (EVs) are nanoscale biological lipid-bilayered vesicles released spontaneously during cellular processes are released without a functional nucleus [19,20]. EVs, which serve as a critical messaging way in biological systems, have the ability to regulate functions of receiver cells via the rich molecular content they carry [21]. According to the recommendations from the International Society for Extracellular Vesicles (ISEV), the term ‘EV’ is accepted as an umbrella term that includes heterogenous structures. EVs are classified operationally based exclusively according to structural properties; such classification takes into account criteria such as particle size (micro EVs < ~200 nm; macro EVs > ~200 nm), concentration, biological indicator signature, or cell/tissue origin [22].

In biogenesis-based nomenclature, the term ‘exosome’ refers to vesicles of endosomal origin, while the term ‘microvesicle’ refers to vesicles formed by budding from the plasma membrane. However, for these nomenclatures to be valid, direct proof of origin is required through experimental methods (e.g., immunolabeling, electron microscopy) [22]. In plant-based vesicle studies, because this origin is often difficult to definitively determine the standard terms ELN or PELN are used. These terms are preferred to emphasize the structural and functional similarities without making any direct inference about biogenesis. Plant EVs can also be subclassified according to the origin of the plant tissue. The subclassification is conducted according to sources including fruit, leaf, root, seed, or apoplast fluid of the plant biogenesis of the vesicles in each of the tissues is proposed to stem from cell structures including multivesicular bodies, exosome-positive organelles, or the vacuolar pathway [19]. Direct visualization of such proposed origins remains for the most part limited and with the need for future research to employ the use of high-resolution imaging methods [22].

### 2.1. Bioactive Cargo Composition of Plant-Derived Exosome-like Nanoparticles

#### 2.1.1. microRNAs

**Figure 1:**
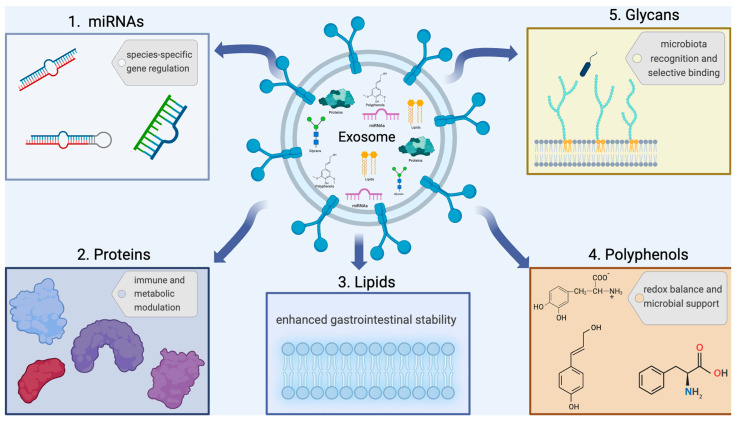
Bioactive cargo composition of PELNs. miRNAs taken up through food and carried by exosomes can modulate gut microbiota and the host’s physiology [23,24]. PELNs carry polypeptides like annexins, heat-shock proteins, aquaporins, and antioxidant enzymes that are involved in vesicle biogenesis, signal transduction, and immune modulation [25,26]. Polyphenols produce SCFAs and phenolic metabolites through biotransformation by the gut microbiota [27]. Polyphenols exert prebiotic effects by suppressing pathogens while increasing beneficial bacteria [28]. PELNs have a unique lipid composition rich in PC, PE, PA, DGDG, and sphingolipids. This structure determines stability, cellular uptake, and biodistribution [29]. Surface glycans play a role in host-microbe interaction by affecting target cell selectivity and cargo transport [30].

Epigenetic modulation of transcriptional output is a type of crosstalk between the surrounding environment and the interpretation and synthesis of genetic data embedded in nucleic acids. Therefore, we hypothesize that activation of the microbiota via applying functional elements in eggs additionally modulates miRNA activity in the liver. miRNAs are sRNA targets specified by the genome that inhibit the transcription of intended genes [23]. Mature miRNA attaches to the three main untranslated sequences (3′-UTR) of the target, promoting its degradation and stalling protein synthesis. This mechanism ultimately leads to the suppression of a specific gene’s activity. Since this association is characterized by non-complementary sequences, a single miRNA can regulate hundreds of target genes. Epigenetic control factors such as miRNAs affect the protein synthesis rates from the mature mRNA without altering the underlying genetic code. The integration of epigenetic mechanisms with the effect of miRNAs may lead to changes in the transcriptional profile by forming an miRNA-driven epigenetic regulatory loop [23]. As shown in Figure 1, miRNAs serve as fundamental modulators and key drivers of many important cellular processes, such as the timing of maturation, nerve cell identity, cell loss, lipid metabolism, and proliferative growth. Host miRNAs can suppress or promote the proliferation of designated microbial species found in the intestine [23]. Some miRNAs are closely linked to specific microbial communities along with particular bacterial groups. The internalization of host miRNAs by bacterial cells has been documented as a mechanism that influences the survival or inhibition of specific microbes. Exosomes, which are fundamental biological carriers for systemic miRNAs, promote intercellular communication and migrate across the blood–brain barrier to integrate into target cell membranes. Including the presence of both botanical- and animal-derived nutrients in our daily food intake enables the horizontal movement of miRNA molecules into the human gut, where they undergo sequestration and perform various biological functions. The first discoveries of miRNA inter-realm relationships include miR-168a from rice, which potentially lowers the LDL levels in the liver. The administration of plant-derived diet-enriched miR-146a to mice with miR-146a deficiency has shown improvements in gut microbiota and gut health [24].

#### 2.1.2. Proteins

Proteins in PELNs, as shown in Figure 1, play critical roles in cellular dynamics, providing a foundation for future therapies. PELNs exhibit different protein profiles encompassing diverse functional categories engaged in stress adaptation mechanisms, signal transduction, various intracellular mechanisms, and they show diversity among plant sources. The annexins, heat shock proteins, and aquaporins defined in PELNs play a role in the formation of intracellular vesicles, the integration of membranous surfaces and regulation of immune homeostasis [25]. Citrus-derived vesicle fractions are notably replete with Patellin-3-like, clathrin heavy chain, and 14-3-3 proteins, alongside substantial levels of glyceraldehyde-3-phosphate dehydrogenase and fructose bisphosphate aldolase. The existence of membrane-bound aquaporin channels is a characteristic feature of nanovesicle fractions. Additionally, a diverse array of hydrolytic and redox enzymes carried within vesicles obtained from citrus fruits potentially mediate various physiological activities. Catalase, a robust antioxidant system comprising superoxide dismutase, peroxidase, ascorbate peroxidase, and glutathione reductase family enzymes is highly expressed in all of the vesicle preparations [26]. Ginger PELNs typically contain intracellular proteins, including structural elements like actin and various proteolytic enzymes and several membrane proteins (e.g., aquaporins and chloride channels) [31]. Aloe PELNs have been found to contain HSP70, glutathione S-transferase, annexins, adenosylhomocysteinase, and GAPDH [29,32].

#### 2.1.3. Polyphenols

Polyphenols stand as a prominent category of plant-derived bioactive compounds within human nutrition and are increasingly recognized as new prebiotics, owing to their ability to optimize gastrointestinal health and counteract dysbiotic conditions. As shown in Figure 1, polyphenols undergo microbial biotransformation in the gut, converting into low-molecular-weight phenolic metabolites that can regulate host physiology and immunological efficacy. Among these metabolites, SCFAs inhibit pathogens by lowering lumen pH and provide nutrients for beneficial microbes [27]. Dietary polyphenols have been shown to promote beneficial bacteria such as *Bifidobacterium* and *Lactobacillus* while simultaneously inhibiting the growth of pathogens like *Clostridium* and *Escherichia coli*. Polyphenols also possess antimicrobial, antioxidant, and anticancer properties. Glycosylated polyphenols that reach the colon undisrupted decompose free-form via hydrolysis by gut microbiota. This process remarkably enhances biological activity and the survival rate of the systems of the probiotics [28]. This provides both health benefits and prebiotic effects [33].

#### 2.1.4. Lipids

The lipidic architecture of PELNs is characterized by a unique profile, predominantly composed of structural lipids, including phosphatidylcholine (PC), phosphatidylethanolamine (PE), phosphatidic acid (PA), and digalactosyl diacylglycerol (DGDG) [25,32,34]. Ginger-derived PELNs contain high levels of PA (35.2%) and DGDG and are selectively internalized by *Lactobacillus rhamnosus*. Grapefruit PELNs are rich in PC and are preferred by *Ruminococcaceae*. The lipidomic profile of aloe PELNs reveals a significant enrichment of glucosylceramide (reaching 40%) and ceramide (Cer, 10%) [29]. Sphingolipids (especially GIPCs) are highly abundant in the PELN membrane and may play a role in signal transduction [30]. PA acts as a signaling messenger within the framework of cellular regulation, including critical processes like membrane fusion and protein binding [32]. As shown in Figure 1, the lipid composition determines the stability, biodistribution, and uptake of PELNs into target cells.

#### 2.1.5. Glycans

Glycans are found in the EV membrane as glycoproteins or glycolipids and play a critical role in host–pathogen interactions. Histo-blood group antigens, such as ABO and Lewis antigens, function as bioactive oligosaccharide chains that can be covalently linked to lipid or protein moieties and are associated with the composition of the intestinal microbiota. Free glycans in secretions such as mucus or milk provide defense by binding pathogens like “trap receptors.” As shown in Figure 1, glycans can influence the target cell selectivity and cargo delivery efficiency of EVs. Additionally, the necessity of glycomics knowledge in personalized medicine approaches is emphasized [30].

### 2.2. Major Edible Plant Sources of Exosomes

#### 2.2.1. Ginger

As a perennial member of the *Zingiberaceae* family, ginger (*Zingiber officinale Roscoe*) stands out as a traditional medicinal plant characterized by a diverse profile of phenolic and terpene compounds [35,36]. The phytochemical composition of the rhizome contains various components such as fatty acids (3–6%), protein (9%), carbohydrates (60–70%), crude fiber (3–8%), ash (~8%), water (9–12%), and volatile oil (2–3%) [35]. Its therapeutic properties are derived from its main phenolic compounds, gingerol (GN) and shogaol (SG) [35,36]. These substances demonstrate versatile biological activities, including anti-inflammatory, antibacterial, immunomodulatory, antitumor and antioxidant activities [16,35,36,37]. Huang-Ge Zhang and colleagues introduced ginger-derived exosome-like nanovesicles (GELNs) into the literature for the first time in 2014 [32,35,36]. Zhang et al. stated that GELNs contain 6-GN and 6-SG, which show anti-cancer and anti-inflammatory features. Moreover, these nanovesicles serve as safe biological vectors for the transport of drugs and sRNAs [36]. Significant biological activity is determined at 30–45% concentration fractions of GELNs that were purified using sucrose density gradients [37]. GELNs have therapeutic potentials of critical importance in a wide range, such as restoration of gut microbiota homeostasis, suppression of inflammation, prevention of hepatotoxicity related to alcohol, prophylaxis of type 2 diabetes, and treatment of colitis-associated cancer [35].

#### 2.2.2. Grape

In one of the studies, purified grape-derived exosome-like nanoparticles (GDENs) extracted from grape juice were subjected to LC-MS/MS analysis. As a result of this proteomic analysis, a comprehensive set of 246 peptides representing 121 distinct proteins was detected in these vesicles obtained from Bobal grape berries [38]. GDENs typically exhibit a particle size distribution between 37 and 380 nm, alongside having a zeta potential spanning from −69.6 mV to +2.52 mV [39]. Transmission electron microscopy (TEM) analyses of GDENs obtained from Kyoho grapes have demonstrated that these nanovesicles have a circular morphology and a bilayer lipid membrane structure [40]. In lipidomic analyses, the presence of components such as PA (53.2%), PE (26.1%), sphingosine (SPH, 83.4%), triglyceride (TG, 7.09%), PC (6.19%), and Cer (1.17%) was identified. These lipid compounds play a role in maintaining the stability and fluidity of the vesicle membranes and in regulating cellular signal transduction mechanisms [40,41]. Proteomic and transcriptomic materials that are transmitted via GDENs provide transcriptional regulation of mediators such as IL-1, IL-2, IL-5 and IL-6 by affecting inflammatory signaling pathways of targeted cells [39]. GDENs, which structurally resemble artificial liposomes, offer high biocompatibility, low immunogenicity, and easy modification advantages in the transport of drugs, siRNA, DNA, and proteins; they can cross the blood-brain interface but cannot bypass the placental barrier [39]. Both in vitro and in vivo evidence indicates that GDENs suppress ultraviolet-induced skin aging through the reduction in epidermal width, preventing the reduction in collagen fiber density, and increasing antioxidant capacity; they also exhibit tumor-suppressive effects in the transport of anticancer agents such as metformin, doxorubicin, and tamoxifen [40,42]. However, when administered orally, dextran sulfate sodium has been documented to offer protection against colitis, being internalized by intestinal stem cells and macrophages and triggering the Wnt/β-catenin signaling pathway [41,42]. These properties demonstrate that GDENs are promising nanocarrier systems for the transport of bioactive compounds, disease treatment, and cosmetic applications due to their natural, biocompatible, and stable structures.

#### 2.2.3. Citrus

In citrus fruits such as lemon (*Citrus limon*), exosome-like nanoparticles are naturally found in the vesicles of the fruit juice, and these structures effectively transport bioactive micronutrients such as vitamin C and citrate to mammalian cells without degradation [43]. Citrus fruits contain the highest levels of carotenoids found in any fruit and a wide range of secondary compounds with important nutritional attributes; these bioactive constituents contribute to the formation of anticancer and nutraceutical molecules exhibiting potent antioxidant, anti-inflammatory, cholesterol-lowering, and anti-allergic activities and are secreted as a plant-derived secretome localized within the apoplast of the juice vesicles; they are packaged within exosome-like nanoparticles [43,44,45]. Citrus fruits, including *Citrus limon*, are among the most commonly studied edible plants for their exosome-like nanovesicles due to their richness in bioactive compounds and antioxidant properties [44]. Exosome-like vesicles isolated from *Citrus limon* (EXO-CLs) are nanoscopic bilayer lipid structures that are morphologically and structurally similar to mammalian exosomes, rich in fatty acids, proteins, and RNAs, and typically have average diameters ranging from 100 to 200 nm [43,44]. These structures exhibit antioxidant effects by reducing oxidative stress and regulating cell differentiation; thus, they become candidates for natural nanocarriers in interspecies communication and functional foods [43,44]. It has been demonstrated that EXO-CLs carry measurable substantial quantities of citrate and ascorbic acid and also contain small RNA fragments (20–30 bp) whose functional significance remains elusive, although specific miRNAs could not be detected [43,45]. Citrus-derived exosome-like nanovesicles, characterized by an average particle size of 154. 5 ± 1.9 nm in size, round or oval in shape, and integrate with target cell membranes via direct plasma membrane fusion or endosomal membrane fusion after endocytosis, exhibiting cross-kingdom effects and facilitating RNA-mediated interaction (RNAi) by effectively transporting sRNAs to target cells [46]. Additionally, the bioactive contents of citrus-derived exosome-like nanovesicles, which include flavonoids, limonoids, and ascorbic acid, collectively underpin their antioxidant and anti-inflammatory potential due to their non-toxic and biocompatible nature; these structures are promising candidates for natural nanocarriers for drug and nutraceutical delivery [44].

#### 2.2.4. Broccoli

Broccoli is an edible plant rich in bioactive phytochemicals, and exosome-like nanoparticles derived from it exhibit significant biological activities [47]. Studies have shown that these nanovesicles reduce inflammation and uphold intestinal immune homeostasis by increasing the stimulation of the adenosine monophosphate-activated protein kinase (AMPK) signaling axis and enhancing dendritic cell (DC) tolerance in animal colitis models [47,48,49]. Additionally, it has been determined that PELNs can transport fluorescent molecules to keratinocytes due to their high lipophilic properties and that these properties have potential for transdermal applications in tissue regeneration [47]. Broccoli-derived nanovesicles demonstrate a preventive effect against colitis via the inhibition of phosphorylation of the MTOR/S6 kinase (S6K) pathway in parallel with AMPK activation, thereby exerting suppressive effects on inflammatory factors (IFN-γ, interleukins, TNF-α) [48,49]. These nanovesicles are rich in sulforaphane content, in addition to their antiproliferative and antioxidant properties; HPLC analysis has shown that sulforaphane is more concentrated in these nanocarriers than in microparticles [48,49]. The mean particle sizes of broccoli exosomes range from 75 to 400 nm, with an average of 146.7 ± 7.2 nm. Their morphology and density have been confirmed by TEM and nanoparticle tracking analysis (NTA) [50,51]. Western blot analyses have demonstrated that exosome markers such as CD63, CD9, and TSG101 are highly expressed [50]. These vesicles resist RNase enzyme and gastrointestinal digestion, enabling the miRNA they carry to be internalized by Caco-2 intestinal cells and affecting cell viability [51]. Additionally, it has been observed that broccoli exosomes labeled with PKH26 are internalized by macrophages and L929 fibroblast cells via endocytosis. It has been determined that they enhance the transition of macrophages from the M1 to the M2 phenotype by suppressing NF-κB-mediated proinflammatory signaling and increasing pro-regenerative cytokines, including TGF-β and IL-10 [50]. Thus, it is suggested that broccoli-derived exosomes have a potential for stimulating tissue regeneration by abilities such as removing reactive oxygen species (ROS), reversing inflammation, and regulating the immune microenvironment [47,50].

#### 2.2.5. Turmeric

Turmeric is a perennial plant belonging to the ginger family and has anti-inflammatory, anti-cancer and cardiovascular beneficial features. Curcumin serves as the primary polyphenolic constituent of turmeric. It is a natural polyphenol and shows antioxidant, anti-inflammatory, anti-angiogenic and anti-cancer effects [52]. Turmeric-derived exosome-like nanoparticles are natural nanovesicles characterized by a complex molecular architecture comprising proteins, lipids, nucleic acids, and small-molecule metabolites, possessing remarkable biocompatibility and safety properties [53]. These nanovesicles were isolated by ultrasonic centrifugation and sucrose gradient centrifugation, exhibiting mean diameters spanning the range of 178 to 183 nm and zeta potentials measured between −17.6 and −21.7 mV [52,53]. TEM analyses have revealed that these structures have a typical teacup-shaped or hemispherical morphology and resemble exosomes derived from mammalian cells [52,53]. Turmeric-derived exosome-like nanoparticles yielded approximately 1.71 ± 0.176 mg per gram of fresh turmeric and demonstrated their richness in bioactive molecules by preserving approximately 36.4% of the total curcumin in turmeric rhizomes [53]. In lipidomic analyses, 615 different lipid types were identified in these nanovesicles, the main ones being PE, PC, phosphatidylinositol (PI), DGDG, monogalactosyl diacylglycerol (MGDG), PA, and TGs [52,53]. Protein composition analyses have shown that approximately 70% of proteins in turmeric-derived nanovesicles are involved in metabolic pathways and secondary metabolite biosynthesis [54]. When administered orally, turmeric-derived nanovesicles exhibit preferential accumulation in inflamed gut regions, enhancing the synthesis of tight junction proteins, consequently reinforcing the integrity of the intestinal barrier. It has been determined that turmeric-derived nanovesicles alleviate colitis symptoms by modulating proinflammatory cytokines and inhibiting the NF-κB pathway and regulating the microbiota by reshaping the macrophage phenotype [54,55]. Additionally, an increase in the relative abundance of *Akkermansia*, *Lactobacillus*, Clostridia_UCG-014, and *Bifidobacterium* species was observed after turmeric-derived nanovesicle treatment [54]. It has been reported that curcumin reduces the inflammatory response by inhibiting the activation of NF-κB, IL-8, NOS, PGE2, and COX-2, thereby alleviating symptoms in diseases such as osteoarthritis [56]. Curcumin-loaded exosomes have high bioavailability, solubility, and safety profiles. Therefore, they have nontoxic therapeutic potentials. Also, they significantly increase plasma concentration and bioavailability of curcumin [57].

#### 2.2.6. Aloe Vera

The leaves and roots of aloe vera, which contains approximately 550 species, contain rich pharmacologically active phytochemicals and various phenolic compounds [58]. Aloe-derived ELNs are noteworthy as systems that carry their bioactive compounds. It has various advantages, including reduced immunogenicity, economic viability, and streamlined industrial synthesis [58,59]. In one of the studies, indocyanine green was loaded onto aloe vera gel-derived ELNs, demonstrating that these nanoparticles destroyed melanoma cells and inhibited the growth of a tumor. Thereby confirming that they have potent targeted drug delivery and transdermal skin permeability ability. According to NTA analysis, the average particle concentration of the specimens is determined as 5.3 × 10^11^ and 4.1 × 10^11^ particles/mL, respectively. In the extent of physicochemical characterization, dimensional and electrical features are reported as 190 nm and 160 nm, and −14.59 mV and −24.26 mV [58]. Hypotonic stress caused by ICG has led aloe cells to excrete more ELNs carrying long-chain sterol esters, PC, PI, and Cer. These vesicles, which have a cup-shaped morphology, they also have a compounding richness in long-chain lipids [59]. Aloe vera-derived nanovesicles can function as oxygen carriers that enhance tissue repair in addition to their ability to oxygenate the hypoxic environment in wound areas [60].

Edible PELNs have been successfully isolated from diverse biological origins, including ginger, grape, citrus, broccoli, turmeric, aloe vera, tomato, apple, green tea, and rice, highlighting the broad diversity of natural nanovesicles with bioactive potential.

### 2.3. Differences Between Plant EVs and Mammalian EVs

Although PELNs are analogous to mammalian EVs in terms of structure and function, they exhibit distinct differences in terms of biogenesis, composition, targeting, and biological functions [61,62]. Both systems secrete exosome-like vesicles, thereby resulting in the fusion of multi-vesicular bodies (MVBs) with the plasma membrane; however, plant cells lack the endosomal sorting complex (ESCRT-0), and instead, plant-specific proteins such as FREE1 and TOL perform a similar function [61,63,64,65,66]. Additionally, the maturation of MVB in plants occurs through the transformation of clathrin-coated networks within the Golgi stack matrix, which proceeds similarly to the role of early endosomes in mammals [64]. PELNs have a lipid bilayer membrane structure and transport bioactive cargo such as proteins, mRNAs, lipids, and phytosterols (e.g., β-sitosterol); in terms of lipid composition, they contain molecules specific to plant membranes such as PA, PC, PE, and glycolipids. In contrast, mammalian EVs are characterized by cholesterol, sphingomyelin, sphingolipids, cytokines, and growth factors [62,66,67]. Glycosyl inositol phospholipids (GIPCs), which are found only in plants and fungi, are present specifically in *Arabidopsis* EVs, whereas this structure is not observed in mammals [65]. Although structurally similar, PELNs generally have a slightly larger particle size than mammalian EVs [67]. Mammalian EVs can generally be easily isolated from fluids such as blood, milk, and urine, whereas the isolation of plant EVs requires more complex procedures due to the low amount of extracellular fluid in plant tissues [65]. However, PELNs derived from plants are much more efficient, scalable, have a lower cost and have lower immunogenicity in terms of production [62,67]. Additionally, PELNs stand out for their high biocompatibility, low cytotoxicity, and target tissue-specific endocytosis capabilities [61,62]. TET8/TET9 proteins from the tetraspanin family in plant EVs function as homologs of CD9, CD63, and CD81 which are found in mammalian EVs and play a decisive role in PELN biogenesis [61,65].

**Figure 2:**
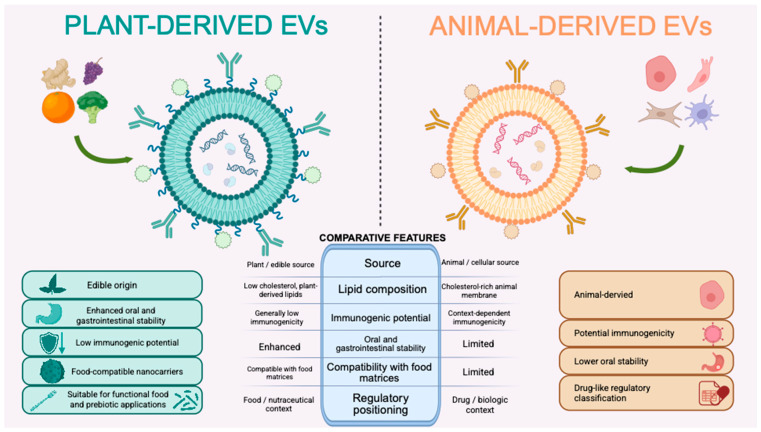
Comparative features and translational advantages of PELNs compared to animal EVs. PELNs differ from mammalian EVs in biogenesis, molecular composition, and targeting while offering major translational advantages, including animal-free sourcing, ethical acceptability, low immunogenicity, and high biocompatibility [61,62]. Readily extracted from edible and agricultural plants like ginger, aloe, grapes, citrus fruits, and broccoli, PELNs are scalable, environmentally sustainable, and suitable for oral delivery, positioning them as next-generation biologically effective nanocarriers and prebiotic platforms [65,67].

In summary, as shown in Figure 2, there are some structural and functional similarities between PELNs and animal EVs. However, there are significant differences in the perspective of protein profile, immunogenic properties and lipid compositions. Mentioned differences highlight PELNs as an applicant for both therapeutic and functional food applications.

## 3. Isolation, Characterization, and Stability in the Digestive System

Ultracentrifugation uses high-speed rotation to fractionate vesicles based on their size-to-density ratios. This technique is considered the gold standard for exosome isolation [67,68]. The general procedure involves a step-by-step isolation guide with different centrifugal forces at different levels. The first step is removing cells and large debris using low-speed centrifugation steps (300–400× *g* 10 min). It is followed by sequential centrifugation at 2000× *g* and 10,000× *g* to remove cellular debris. In the final step, centrifugation is performed at 100,000–200,000× *g* to pellet exosomes or PELNs [67,68]. Although ultracentrifugation protocol is a standard approach, it comes with several technical disadvantages. Notably, the risk of damage and aggregation in vesicle morphology from dense mechanical stress can affect the reliability of obtained data. Additionally, extended processing time and low throughput capacity prevent the method from becoming an efficient option for large-scale application [69,70]. To overcome these mentioned limitations and maintain structural integrity of vesicles, an alternative technique, size exclusion chromatography (SEC) is extensively preferred [70]. SEC is a technique that separates fractions by size as they pass through a column with porous beads. It is also gentle, reproducible, and preserves biological activity [67,69]. Additionally, it is industrially feasible and economical, making it suitable for therapeutic applications [67]. On the other hand, polymer-based precipitation offers an alternative for the isolation of exosomes and PELNs [70]. The usage of polymeric reagents such as polyethylene glycol (PEG) is based on the exclusion of water molecules in the environment. This process promotes controlled aggregation by reducing the solubility of exosomes. These vesicle aggregations that occur are efficiently reobtained using low-speed centrifugation after the incubation stage [68]. PEG 6000 is also known for its ability to trigger membrane fusion aside from precipitating exosomes selectively. This specific characteristic is especially and actively used in the development of crossbreed PELNs. The respective method facilitates the integration of vesicles from different plant species into hybrid structures [70]. However, PEG precipitation may cause aggregation. This is an important disadvantage that negatively affects the purification level of obtained samples. Therefore, additional steps are recommended for applications requiring high purity [69].

In direct relation with the data mentioned in Table 1, each isolation method has its own advantages and drawbacks in terms of efficiency and purity criteria. Due to these reasons, generally hybridized or sequential combinations of these methods are used to reach maximum efficiency according to the targeted analysis sensitivity.

The complete characterization of PELNs requires a comprehensive analyzing process that targets understanding the physical and chemical nature of particles. Within that period, the main goal is determining morphological structures, size distributions and surface characteristics of nanoparticles. These examinations, conducted with multifaceted approaches, have critical importance for verifying the biological functionality of vesicles [16,71]. Morphological characterization of PELNs is fundamentally conducted by using TEM, scanning electron microscopy (SEM) and atomic force microscopy (AFM) methods. These methods are widely employed to define the surface and structural characteristics of the vesicles [16,20,71,72]. Due to its high resolution, TEM is able to examine morphological details of PELNs at subcellular levels. With this technique, spherical or cup-shaped structures of vesicles have been visualized [16,49,71]. Dynamic light scattering (DLS) and NTA techniques are prominent in determining size and surface charge [72]. DLS is a method that determines the hydrodynamic diameters of suspended particles based on Brownian motion; they are non-invasive and highly accurate. However, due to resolution limitations, it can cause some complications in the analysis of heterogeneous samples. NTA is currently preferred for more reliable measuring of the particle size profile and number density of the 10–2000 nm range [16,49,71]. NTA technology is based on the principle of individual tracking of every particle that moves under a laser beam. Through this method, both their concentration and hydrodynamic diameter can be calculated simultaneously. Also, the low sample volume requirement of the process makes this method a highly efficient and rapid analytical tool [16,49]. Zeta potential analysis, that determines surface charge of nanovesicles is a critical parameter for understanding colloidal stability and molecular interactions of a system. In the literature, values typically ranging from −30 mV to +30 mV indicate high stability [71]. To determine the biochemical and lipid composition of PELNs, Fourier transform infrared spectroscopy (FTIR) and sulfo-phospho-vanilin (SPV) tests are applied. FTIR facilitates insights into the functional molecular groups. On the other hand, the SPV assay allows the quantitative determination of the total lipid composition [16,72]. This comprehensive approach completely determines the physical and biochemical structure of PELNs. Thus, it presents a standard reference for understanding the biological functions of vesicles.

Although the studies about PELNs gained momentum, there is no developed standard protocol to measure the purity of these structures. When the common characterization methods are considered in existing literature, a minimum criteria set can be recommended for a complementary evaluation. In this framework, it is important to analyze the basis of morphological structure via TEM, size distribution via NTA, surface charge via zeta potential, and protein residue or pollutant via nucleic acid amounts. Although these mentioned parameters do not give any ultimate evidence, complementary evaluation will prepare an applicable and reliable basis for the verification of PELN purity in different research ecosystems.

## 4. Prebiotic Functions of Plant-Derived Exosome-like Nanoparticles

### 4.1. Crosstalk Between Plant-Derived Exosome-like Nanoparticles and Gut Microbiota

PELNs have a multidiverse effect on mechanisms in both direct contact and indirect ways on gut microbiota. They restructure the microbial ecosystem by stabilizing the immune system and enhancing the integrity of the epithelial barrier and shape bacterial diversity directly [73,74]. PELNs play a key role in maintaining intestinal mucus protection. These nanoparticles enhance the production of beneficial bacteria that increase mucus production, whereas they inhibit activity of harmful pathogens that damage mucus barriers [16,73]. PELNs manage communication between plants and microorganisms by safeguarding the transportation of protein and RNA molecules. Within this period, plant miRNAs play an active role in maintaining intestinal microbial homeostasis by interfering with gene expression of both human and bacteria cells [16,74,75]. PELNs from ginger, grape, tea, and lemon transmit bioactive compounds that they contain by resisting tough conditions of the digestive system. These nanoparticles suppress inflammation and enhance tissue regeneration. Also, they help to maintain microbial balance by strengthening intestinal barriers [16,75]. Lipids, metabolites, proteins and RNAs in the structure of PELNs work in harmony. Lipids manage microbial control by enabling cells to uptake these molecules easily. Simultaneously, miRNAs help protect probiotic species and enhance microbial diversity by programming growth and metabolism processes of bacteria directly [74,75]. PELNs play an active role in preserving intestinal homeostasis by their anti-cancer and anti-inflammatory features. It especially helps the digestion system to return to its healthy microbial balance by alleviating dysbiosis. Orally administered PELNs are internalized by intestinal epithelial cells by directly targeting inflamed colon tissue in colitis models. These particles located around the cell’s cytoplasm and nucleus, manage regeneration processes by building crosstalk communication between host cells and gut bacteria [74,76,77]. Garlic-derived PELNs inhibit reproduction of pathogenic microorganisms such as *Helicobacter* and *Escherichia*-*Shigella* while increasing the beneficial bacteria populations such as *Lachnospiraceae* in the gut. Due to this microbial regulation, SCFA production is enhanced, and also systemic inflammation is decreased [75,78]. GELNs are internalized by intestinal macrophages and also bacteria. Notably, the lipids in their structures act as a signal that increases uptake by *L. rhamnosus* [16,35]. Furthermore, outer membrane vesicles from *A. muciniphila*—stimulated by garlic-derived ELNs—have been shown to reverse disease progression using a dietary-induced model of type 2 diabetes [78,79].

### 4.2. Modulation of Microbial Composition by Plant-Derived Exosome-like Nanoparticles

PELNs inhibit pathogens by modulating the gut microbiota via enhancing the beneficial bacteria growth. *Actinidia arguta*-derived PELNs increased *Klebsiella*, *Acinetobacter*, and *Lactobacillus* abundance while decreasing *Proteus*, *Escherichia*/*Shigella*, and *Paeniclostridium* [73,75]. Garlic-derived PELNs promoted *A. muciniphila* proliferation in high-fat diet mice, and lemon-derived vesicles enhanced bile resistance of *L. rhamnosus* and *Streptococcus thermophilus*, protecting against *Clostridium difficile* infection [16,73,74]. These nanovesicles suppress pathogens such as *Porphyromonas gingivalis* through interactions of PA on ginger PELNs with bacterial surface protein HBP35 and by inhibiting Msps expression and tRNA integrity in S.24-7 bacteria. They also increase probiotic resilience by protecting *L. rhamnosus* GG from bile stress [16,74,75]. PELNs enhance microbial diversity and abundance by elevating probiotic levels and reducing opportunistic pathogens. Lipids such as PC and PE contribute antioxidant, anti-inflammatory, and anti-colitis properties while mediating microbial uptake and maintaining homeostasis [16,80]. GELNs are primarily internalized by *Lactobacillaceae* and upregulate IL-22 by targeting *L. rhamnosus* genes, thereby strengthening the intestinal barrier and alleviating colitis [35,80]. In DSS-induced colitis and high-fat diet models, PELN treatment increased SCFA-producing bacteria such as the *Lachnospiraceae NK4A136 group*, *Lactobacillus*, and *Butyricicoccus*, while decreasing *Escherichia–Shigella* and *Enterococcus*. Tea leaf-derived ELNs suppressed bacterial invasion and reinforced the epithelial barrier [75,77]. Medium- and high-dose EXO-CLs restored microbial balance by increasing *Bacillota* and reducing *Bacteroidota*, while GELNs enhanced barrier function via miR-7267-3p targeting of *ycnE* in *L. rhamnosus* [35,75,78].

### 4.3. Roles of Exosomal miRNAs and Glycans in Microbe-Host Signaling

PELNs contain diverse nucleic acids-including miRNAs, mRNAs, non-coding RNAs (ncRNAs), and sRNAs—that enable inter- and cross-kingdom communication by modulating microbial and host gene expression. Plant-derived miRNAs, typically 22 nt non-coding molecules, can suppress mRNA translation, trigger degradation, and regulate specific genes, thereby shaping gut microbial ecology and inflammatory signaling [16,75,80]. *Rehmanniae radix*-derived PELNs inhibit *E. coli* biofilm formation via miRNA-7972, while ginger PELNs rich in PA are preferably internalized by *L. rhamnosus* [35,73]. These miRNAs can be absorbed by bacteria and regulate microbial gene expression; some, such as miR-2911 from honeysuckle exosomes, even target viral genomes like SARS-CoV-2 and suppress replication [16]. Accumulated studies demonstrate that PELNs transmit signals across species through bioactive molecules (lipids, proteins, and nucleic acids) that alter microbiota composition and alleviate colitis [16,80]. The miR-44 and miR-54 molecules found in tea-leaf-derived nanoparticles (TLDENs) protect gut barrier function. Simultaneously, miR-7267-3p derived from ginger enhances barrier integrity and also triggers the production of IL-22, which is critical for the immune system by regulating the *ycnE* gene in *L. rhamnosus* [35,77]. The miRNAs that EXO-CLs contain have comprehensive control mechanisms on bacterial genetic processes. These molecules, notably crt-miR166a, crt-miR168 and crt-miR171, have the potential to directly change the action of microbial genes by interfering with bacteria miRNAs [74,78]. Ginger and turmeric ELNs are mainly internalized by Bacteroidales S24-7 and Lactobacillaceae, while garlic and grapefruit ELNs target la and Ruminococcaceae [75,78]. The PA localized to the ginger-derived vesicles bind to the HBP35 protein of *P. gingivalis*, exhibiting an antibacterial effect. These complex miRNA- and lipid-mediated interactions highlight PELNs as potential therapeutic modulators of the microbiome, though further studies are required to optimize their clinical application [16,73,74].

### 4.4. Short-Chain Fatty Acid Production and Mucosal Immune Modulation

PELNs promote the production of SCFAs via enriching SCFA-producing bacteria after treatment [73,75]. GELN RNAs stimulate *L. rhamnosus* GG to produce I3Ald, stimulating the aryl-hydrocarbon-receptor (AhR) signaling pathway in intestinal lymphocytes, up-regulating IL-22, and alleviating colitis [16,74]. Similarly, PELNs modulate macrophages by increasing IL-10 and heme oxygenase-1 (HO-1) expression and suppressing NLRP3 inflammasome activation, while broccoli-derived PELNs induce tolerogenic CD11c^+^ DCs and maintain immune homeostasis [74,80]. GELNs activate the AhR pathway via I3Ald, enhance IL-22 and tissue repair, and strengthen the mucosal barrier. Lemon-derived ELNs elevate AhR ligands indole-3-lactic acid and I3Ald, induce IL-22, and inhibit *Clostridioides difficile* growth and indole biosynthesis by raising lactic acid levels, reducing fecal pathogen counts [16,75]. Garlic-derived vesicles markedly increase acetic, butyric, propionic, valeric, isobutyric, and isovaleric acids, correlating with higher abundance of *Flintibacter hominis*, *Flintibacter butyricus*, and *Acetatifactor muris* species linked to anti-inflammatory NF-κB suppression [75,78]. SCFAs enhance gut wall health by acting as a fundamental source of energy for colonocytes. Simultaneously, it protects gut barrier strength and helps the immune system to respond in a balanced manner. High-dose citrus-derived EXO-CLs increase SCFA levels and microbial diversity, mitigating high-fat-diet-induced dysbiosis [74,78].

TLDENs and GELNs elevate IL-22 through miR-7267-3p-mediated AhR activation, triggering antimicrobial immunity, tissue repair, and barrier reinforcement [16,35].

### 4.5. Comparative Microbiota Responses to Different Plant Sources

The functional effects of PELNs differ by the plant that they are derived from, their molecular compositions, and the lipid structure on their surfaces. This variability causes every single plant nanoparticle to trigger different responses in the immune system and make a distinctive regulation.

GELNs enhance production of protective and anti-inflammatory factors whereas they reduce proinflammatory cytokines by selectively targeting the colon. They promote epithelial repair via activating AhR-IL-22 signaling pathway and ensure protecting microbial balance by enhancing *Lactobacillus* abundance [16,74]. Garlic-derived vesicles have considerably comprehensive and positive effects on gut health. These compounds significantly increase the population of beneficial bacteria in the body, especially *Akkermansia muciniphila* [75,78]. Citrus-derived EXO-CLs restore high-fat-diet-induced dysbiosis by elevating *Bacillota* and reducing *Bacteroidota* ratios while enhancing the expression of the junctional markers ZO-1 and Occludin [75,77,78]. TLDENs suppress bacterial invasion and enhance mucosal barrier repair, while grape-derived vesicles alleviate DSS-induced colitis and normalize microbiota composition [75,80]. Broccoli-derived PELNs activate AMPK and induce tolerogenic DCs, maintaining immune homeostasis and preventing inflammation [80]. Blueberry- and wheat-derived PELNs exert antioxidant and regenerative effects; blueberry vesicles reverse endothelial inflammation, whereas wheat vesicles stimulate fibroblast proliferation and collagen synthesis [74]. Among all of the sources, ginger-derived PELNs were the most extensively studied and the only ones tested in a clinical trial for IBD therapy (Clinical Trial ID: NCT04879810), confirming their oral stability and targeted colon delivery [16,80].

Overall, these comparative findings reveal that different plant sources produce vesicles with shared anti-inflammatory and barrier-protective activities but distinct microbial and immunological signatures.

## 5. Applications in Functional Foods and Synbiotic Systems

PELNs are recognized for modulating the functions of pathogenic or gut microbial ecology. Lemon-derived nanovesicles have been reported to exhibit the ability to suppress *Clostridioides difficile*, which causes diarrhea and pseudomembranous colitis, and this effect was mediated by increasing the survival of *S. thermophilus ST-21* and *L. rhamnosus* GG [48]. At the molecular level, PELNs carry small RNAs and proteins that are transferable to probiotic vehicles, enhancing their ability to adhere to intestinal epithelial cells and increasing the expression of genes involved in the synthesis of AhR receptor ligands [49]. This biological process firstly enables the AhR signaling pathway to be activated. This activation leads to the increase of IL-22 in the body. These changes play a critical role in maintaining protection of intestinal barrier integrity and enhancing defense mechanisms [48]. Selective absorption of GELNs by *L. rhamonus* GG starts a biochemical chain. This period I3A where its production is promoted supports the AhR activation and strengthens the work of the mechanism [75]. Polyphenols in tea optimize the gut ecosystem. They strengthen the digestive system’s health by reducing potential harmful bacteria populations, whereas they selectively increase beneficial species [48]. PELNs supply metabolic balance and also form protective shields for beneficial bacteria. Their ability to selectively support beneficial species, whether inhibiting pathogens such as Clostridium, shows that these compounds are more efficient “smart regulators” than classical prebiotic supplements [48,49]. Studies show that nanovesicles derived from lemon and tomato take complementary roles in probiotic bacteria. Lemon nanovesicles provide protection via optimizing the resistance of LGG, whereas nanovesicles derived from tomato and ginger directly promote the increasing populations and production of beneficial metabolites [75]. Cooperation between PELNs and probiotic strains increases the survival rates dramatically in pathogenic infection. The fact that the 11 probiotic and EXO-CL combinations reduce the mortality rate by 40% and that this effect is supported by different strains and provides mounting evidence that these binary combinations would be the “gold standard” in gut health treatments [75]. Either the probiotic enlargement capacity of nanovesicles that carry specific miRNA (gma-miR396e) or the ability of PELNs to support the Lactobacillaceae family shows that this synergy is critical for gut health. These findings reinforce that PELNs act as a “smart fuel” for beneficial bacteria [81]. PELNs can be determined as a “new generation tool” that enhances the vitality of probiotics and biological outputs. Due to these features, they are prominent as the key raw materials in the development of high performance synbiotic formulations that support gut health.

### Stability During Food Processing and Storage

High resistance that PELNs exhibit against digestive enzymes helps them to reach the target points in the body without disruption. These nanoparticles that are safe and bioavailable due to their nature are prominent as a new-generation biocarrier through their ability to triggering a weak immune response [16]. The application of both ExoQuick and ultrasequestration exhibits a gold standard approach in PELN separations. This method prepares the ground for the next therapeutic usages using PELNs, including those derived from ginseng, to obtain purer and more stable forms [16,82]. The ability of PELNs to structural endurance and tissue penetration allows them to establish a communication network at the cellular level. These particles, which can directly show regenerative effects on the sites of injury, play a strong regulatory role in modern medicine because the risk of rejection by the immune system is low, and they can overcome difficult biological barriers [16,17,83]. Zeta potential is one of the most important pieces of evidence that represents the stability of PELNs. Accurate analysis of this value guarantees that nanoparticles stay without aggregation; thus, it guarantees that the intended therapeutic functions are performed successfully [16,82]. The study by Buranasukhon et al., verified the stability of Pluchea indica leaf nanoparticles via zeta potential. These particles preserved their negative charge, eliminated the risk of clumping, and offered long-term stability by not being affected by the changes in time and temperature [82]. Morphological and physical features of PELNs are resistant to tough pH conditions. Tests conducted in simulated gastric and intestinal fluids proved that these nanoparticles can protect their size and surface charges and maintain their function without being degraded along the digestion [82,83].

The study by Kim et al. indicates that 4 °C may be more advantageous than −20 °C for protein protection while preserving ELNs. It was concluded that storage conditions at 4 °C are a more efficient strategy for preserving the content of vesicles. When cellular uptake rates of freshly harvested and freeze–thawed leaf ELNs are compared, it is found that repeated freeze-thawed loops trigger aggregation; thus, the efficiency of cellular internalization is decreased [82].

The results of the study by Hwang et al., which compared the effects of freshly harvested sweet potato energy nitrogen and sweet potato energy nitrogen stored for one year with alkaline phosphatase activity in MC3T3-E1 osteoblast cells, revealed that sweet potato energy nitrogen maintained alkaline phosphatase activity and increased osteoblast proliferation and differentiation even after one year in storage at −80 °C, indicating that there was no significant change in the composition of sweet potato energy nitrogen [82].

Different size and charge changes were found in the studies conducted on the stability of GELNs in the digestion system. Zhang et al. draws attention to the stability of the system via stating mild reduction in size and pH-based charge changes in the stomach and gut simulations. After all, Zhuang et al. stated that the mean size of GELNs, approximately 294,1 nm, increased over 1000 nm in the gut environment. Both studies verified that surface charge directs positively in the stomach acidity while negatively in the gut environment, and these surface charge changes were consistent with the natural charges of the solutions [83]. These findings show that the lipid bilayers of PELNs supply structural stability that gains endurance against environmental changes and protects their content [16].

Lyophilization of PELN supplies durability during extended storage and also reduces the possibility of product damage during transportation and storage [16]. The conducted studies determined that specific PELN subgroups reduced surface charges via separation or aggregation; thus, they became more resistant to gastrointestinal digestion. High structural stability of PELNs in the gastric and intestinal environment supports the critical importance in terms of the oral administration strategies [37].

Preserving the stability of PELNs constitutes a critical obstacle in especially in the terms of determining suitable storage conditions. The method of preserving by freezing at temperatures below the reactivity threshold, which is common in the literature, can threaten particle integrity because of aggregation risks as a result of repeated freeze–thaw loops [37]. Cryoprotectans such as glucose and trehalose minimize freezing-induced damages via forming a preserving layer around the PELN during lyophilization [37,84]. Additionally, during storage, morphological features, functional capacities and biomolecular compositions of PELNs can undergo various structural transformations [84]. Besides cryopreservation to protect PELNs, methods such as lyophilization and spray drying are also used. The cryopreservation method is a storage process used to protect the functional structure of the particles in very low temperatures with the help of special preservatives [84]. Cryoprotectants are divided into two categories according to their permeability: permeable types (glycerol, DMSO, ethylene glycol) can pass through the cell membrane, while impermeable types (sucrose, mannose, trehalose) prevent ice crystal formation by forming hydrogen bonds with water. In one study, cryopreservation at −80 °C with trehalose (6–10%), DMSO and 30% glycerol, or without a cryoprotectant, was found to maintain extracellular vesicle concentrations for up to 6 months; impermeable trehalose was reported to be the most effective option in terms of safety [84]. Preservation methods for maintaining the biological activity of PELNs, facilitating their transport and clinical applications, need to be investigated, and today the most suitable environment for protecting diverse exosome samples is long-term storage at −20 °C and −80 °C [16,19,82,84].

## 6. Preclinical and Clinical Applications and Translational Challenges

In recent years, the potential of edible PELN for intestinal health and microbiota regulation has been systematically investigated in a growing number of preclinical and a limited number of clinical studies. These studies aim to elucidate the bioavailability of PELN isolated from various plant sources, its interactions with target tissues, its effects on immune and inflammatory responses, and its impact on gut barrier integrity and microbiota composition. The following table summarizes the preclinical and clinical studies of edible PELN, detailing the plant source used, experimental model, route of administration, and reported key biological effects, followed by a detailed discussion of the results of each of the studies in the text.

Zhu et al. documented that garlic-derived ELNs encompass 26 lipids, 61 proteins, and 127 miRNAs and inhibit the TLR4/MyD88/NF-κB signaling pathway by targeting Han-miR3630-5p and TLR4. This intervention alleviated DSS-induced colitis symptoms and epithelial erosion and increased tight junction protein expression. Additionally, pretreatment with garlic-derived ELNs restored the abundance of *Lachnospiraceae*, reduced Helicobacter levels, and ameliorated colitis via restructuring the intestinal microbial landscape [85]. Li et al. reported that GDENs overcome the mucosal barrier and are internalized by intestinal stem cells, where they activate the Wnt/β-catenin signaling pathway, increase stem cell proliferation, promote epithelial regeneration, and prevent DSS-induced colitis [80]. As shown in Table 2, Li et al. demonstrated that GELNs were taken up by cellular constituents of the intestinal mucosa, promoting the survival and proliferation of epithelial cells, suppressing proinflammatory cytokines, and restoring the function of the intestinal barrier and microbiota balance in DSS-induced colitis [80]. Li et al. demonstrated that sulforaphane-loaded broccoli-derived ELNs, which selectively target DCs, activate the AMPK signaling pathway, induce tolerogenic DC phenotypes, suppress proinflammatory cytokine production, and maintain intestinal immune homeostasis in experimental colitis [80]. Li et al. concluded that ELNs derived from tea leaves and administered orally were mainly taken up by macrophages, reduced ROS production, suppressed inflammatory cytokines, increased IL-10 production, and alleviated colitis by the restoration of intestinal barrier integrity and gut microbiota diversity [80]. As shown in Table 2, Li et al. reported evaluating the efficacy of GELN as monotherapy or in combination with curcumin in the treatment of colitis. In a human clinical trial (NCT04879810), oral administration of GELN was found to be safe and well-tolerated, exhibiting anti-inflammatory effects both as monotherapy and in combination with curcumin. This GELN-based approach provides therapeutic efficacy without systemic toxicity and has the potential to enhance the bioavailability of curcumin, making GELN a promising oral nanotherapeutic agent for the treatment of IBD [80]. Di Raimo et al. reported that a mixture of PELNs from grapes, blood oranges, mandarins, papayas, and pomegranates exhibited antioxidant and rejuvenating effects on human skin fibroblasts; they reversed H_2_O_2_-induced redox imbalance, enhanced mitochondrial bioenergetics, attenuated mitochondrial superoxide yields, and increased sirtuin-1 levels. Furthermore, the same PELN mixture supported barrier repair and tissue regeneration in a fibroblast wound model and was associated with accelerated wound healing and increased expression of vimentin and matrix metalloproteinase-9 (MMP-9) [86]. However, variations in experimental models, plant sources, and isolation techniques across studies may limit the direct comparability of these findings. As stated in Table 2, Shkryl et al. performed a preclinical evaluation of the effects of exosome-like nanovesicles (GCENs) isolated from grape callus cultures on the triple-negative breast cancer cell line MDA-MB-231. GCEN treatment resulted in a dose-responsive decrease in MDA-MB-231 cellular fitness and survival but did not lead to significant cytotoxicity in normal HEK-293 cells. Fluorescence labeling experiments showed that GCENs were rapidly internalized by cancer cells and accumulated in the perinuclear region. Flow cytometry analysis revealed that GCEN triggered G1-phase cell cycle arrest, which subsequently primed the cells for apoptotic commitment via phosphatidylserine efflux and caspase-3/7 activation. These selective antiproliferative and proapoptotic effects were mediated by trans-δ-viniferin derivatives present in GCENs, and the study suggests that GCENs are natural and safe candidates for anti-cancer nanocarriers [42]. This study, shown in Table 2, evaluated the oral administration of a powder enriched with GDENs in patients with head and neck cancer. As reported by Wu et al., oral administration of GDEN-enriched powder formulations has been investigated as a safe and well-tolerated approach for the prevention or reduction of oral mucositis in patients receiving radiotherapy and/or chemotherapy. These plant-derived nanovesicles have been shown to have low toxicity and high biocompatibility and can facilitate oral mucosal barrier protection without harming normal tissue. The study was conducted as part of a phase I clinical trial (NCT01668849) and demonstrated the potential of maintenance therapy with oral GDEN [87]. Nevertheless, the majority of these findings are derived from preclinical models, and their translation to human settings should be interpreted with caution. In a preclinical study by Zhang et al., edible GELNs were evaluated as oral prophylactic and therapeutic agents in acute and chronic colitis models, as well as in CAC models. Following oral administration, GELNs were effective in the colon, predominantly internalized by the intestinal epithelial-immune interface, and showed no toxic effects. In DSS-induced acute colitis, IL-10^−^/^−^-induced chronic colitis, and AOM/DSS-induced colorectal cancer models, daily oral administration of approximately ~2 mg/GELN (≈0.3 mg/mouse) reduced inflammation, promoted epithelial repair, and decreased tumor formation and tumor burden. This effect was associated with an attenuation in TNF-α, IL-6, and IL-1β levels and an elevation in IL-10 and IL-22 levels. These results demonstrate that GELN, a natural and low-toxicity platform that can be administered orally into the colon, shows promise for the protection and treatment of colitis and colorectal cancer [31]. In addition, differences in dosage, administration routes, and experimental conditions across these studies further complicate the reproducibility of the reported outcomes. As shown in Table 2, a preclinical study by Deng et al. evaluated the oral protective and therapeutic effects of broccoli-derived ELNs in IBD models. Oral administration of broccoli-derived ELNs significantly reduced body weight loss, mucosal inflammation, and colon shortening in DSS-induced colitis; it suppressed pro-inflammatory cytokines such as TNF-α, IFN-γ, and IL-17A while simultaneously increasing IL-10 levels. Mechanistically, broccoli-derived ELNs were shown to act on lamina propria DCs, inducing AMPK activation and, through this pathway, supporting the tolerogenic response of DCs. These results suggest that oral administration of broccoli-derived ELNs has potential as an anti-inflammatory approach in IBD by regulating gut immune homeostasis [88]. Preclinical research by Wang et al. investigated the potential of oral treatment with grapefruit-derived ELNs in a mouse model of DSS-induced colitis. Grapefruit-derived ELNs were shown to be selectively internalized by intestinal macrophages, increasing HO-1 expression in macrophages and reducing colitis symptoms by suppressing IL-1β and TNF-α production. Furthermore, an ELN-based oral administration system was developed, and methotrexate-loaded grapefruit-derived ELNs were administered, showing lower toxicity and higher therapeutic efficacy compared to free methotrexate. In the model details, in the maintenance group, mice were given 10 mg/kg grapefruit-derived ELN daily for 7 days, followed by colitis induction with 2% DSS; in the treatment group, the treatment was administered at a dose equivalent to 5 mg/kg on specific days after the onset of colitis [89]. Yang et al. conducted a preclinical study to determine the clinical viability of ginseng-derived ELNs in a DSS-induced colitis model. C57BL/6J mice with DSS-induced IBD, RAW264.7 macrophages, and LPS-stimulated intestinal epithelial cells were treated orally with ginseng-derived ELNs at doses of approximately 1–2 mg/mL per mouse, and at doses of 5 mg/mL and 10 mg/mL. Treatment with ginseng-derived ELNs reduced pro-inflammatory cytokine levels, suppressed oxidative stress, and significantly decreased intestinal inflammation. Mechanistic analysis revealed that ginseng-derived ELNs inhibited the TLR4 and MAPK signaling pathways and stimulated the p62 Keap1 Nrf2 antioxidant pathway. As a result, the expression of antioxidant enzymes was increased. Ginseng-derived ELNs that strengthen antioxidant defense, achieved multidiverse recovery in barrier integrity, stem cell regeneration, and microbiota variety. They can rearrange the gut microenvironment whilst inhibiting inflammation and oxidative stress. Thus, this makes these nanoparticles an ultra-effective solution for IBD treatment in the preclinical phase [90]. The research performed by Yang et al. shows that bitter melon-derived ELNs are effective in stimulating apoptosis and stopping cell cycles in OSCC cells. These nanoparticles break the chemoresistance and maximize 5-fluorourasil treatment via inhibiting the NLRP3 pathway with the help of the RNA they contain. As a result, these PELNs were evaluated as a promising assisting treatment method that presents anti-tumor and anti-inflammatory effects at the same time [91].

### 6.1. Mechanistic Insights, Limitations, and Translational Considerations

These studies show that a complementary mechanistic framework has not been done yet, although many specific molecular targets and signal pathways are determined to be affected by PELNs. PELNs are assumed to transfer their biologically active charges via internalization by receptor cells via endocytic pathways or membrane interactions. Within that period, pattern recognition receptors or interactions with other surface molecules can trigger cellular uptake or downstream signaling pathways. After the cellular uptake phase, miRNAs, lipids, and other biomolecules in the PELN structure have the potential to modulate pathways which play a role in the fundamental processes such as inflammation, metabolism, and barrier function. Although these mechanisms have not been clarified yet, they form a basis for biological effects which are investigated in different experimental models and emphasize the requirement for deeper mechanistic verification studies.

Although the studies about the effect of PELNs on pathological models such as IBDs, oncology, and tissue regeneration gained momentum, existing data is heterogeneous and essentially at the preclinical level. As shown in Table 2, most of the findings are based on in vitro or animal models which have used different plant sources, isolation techniques and dosage strategies. This methodological diversity complicates the reproducibility of the results and comparableness between studies. Although many studies state promising mechanistic data and therapeutic outputs, the lack of a standardized experimental framework and comprehensive clinical verification blocks the transformation of this information into general knowledge. As a result, although the PELNs show significant potential in different biological processes, obtained results should be evaluated cautiously; more controlled and standardized studies should be conducted for clarification of the translational value of these structures.

Although the literature on the biological potential of PELNs is rapidly expanding, as the data in Table 2 point out, existing findings are limited within the preclinical levels. The lack of clinical studies on humans blocks passing a certain judgment on the application and safety of these particles. Additionally, the lack of standard dosage protocol is an essential obstacle that complicates both the safety analysis and the comparability of different studies with each other. Besides the uncertainty of industrial-scale productions, quality control standards and legislation limit PELNs usage in medicine. All of these factors display a significant discrepancy between the success in the experimental stage and real clinical applications.

In addition to the current translational challenges, several safety-related considerations should also be acknowledged. Although PELNs are generally accepted as low immunogenic carriers, structural sophistication, which plant-derived biomolecules can integrate into the system, should not be ignored. Glycan and lectin-like structures on the vesicle surface have the potential to interact with the immune receptors of the host. Although the current data does not indicate a serious immunogenic risk, especially in oral intake, mild immune modulations or cumulative effects in continuous usage are not eliminated yet. Due to this reason, while the reliability profile and clinical application of potential PELNs are investigated, it is required to focus on the details and provide support with comprehensive experimental studies.

Besides its effects on the immune system, contamination risks need to be discussed meticulously during PELN isolation. Obtaining the particles in question from plants gives rise to the possibility of the involvement of contaminants such as microbial residues, endotoxins, or agricultural chemicals. Although isolation techniques target minimizing these risks, raw material diversity and processing differences can deviate in the final product quality. In this context, for the sake of guaranteeing the safety and results of PELN-based studies at clinical levels, the establishment of strict quality mechanisms is required. This approach represents a crucial threshold for long-term use and medical applications.

### 6.2. Translational Barriers and Current Research Gaps

The ability of PELNs to receptor-based targeting and transmitting their content without lysosomal disruption is the most critical feature that separates this system from traditional methods and increases therapeutic efficiency [92]. To represent the therapeutic success of PELNs, standardization of purification techniques, determination of goal-directed vesicle selection criteria, and comprehensive mapping of effect mechanisms are required. Existing uncertainties represent the fundamental structural challenges of the common usage of these systems [72,92]. Cellular internalization pathways, charge transfer mechanisms, and formations of biological transformations after PELN interaction are among the existing literature that needs a comprehensive explanation [72]. The combination of these nanovesicles that contain natural bioactive compounds with conventional drugs implies the risk of interactions and necessitates the constitution of safe releasing protocols. On the other hand, stability profiles that vary based on plant source cause technical challenges in the integration of PELNs into functional food formulations. When existing regulations are examined, the lack of specific regulatory frameworks for these vesicles on the EFSA side is noteworthy, whereas the FDA administers tough control mechanisms via evaluating these products in medical product classes independently of their origin [93,94]. These vesicles within the European Union can be evaluated the same as in the framework of ‘Novel Food’ in the situation of commercialization as a dietary supplement or functional food compound. However, the lack of standardized safety evaluation protocols and specific terminological determinations causes an apparent regulatory uncertainty in this phase [94,95]. Regulatory inconsistencies that are mentioned constitute a major barrier in front of edible PELNs, that partake in an intersection of functional foods and therapeutic applications to become widespread. The lack of a standardized clinical dosage protocol determination is the fundamental element that limits safety of application and activity, whereas data from ClinicalTrials.gov and the European Registry of Clinical Trials show the inability of clinical applications in this field.

Despite the progress in intended isolation and characterization of mammalian EVs, these techniques do not fit into a standardized framework for PELNs. Today, differential ultracentrifuge based protocols that are dominant in PELN isolation are faced with serious limitations because of the requirements of a high workforce, long periods of operations, and the dependency on advanced equipment [96]. Furthermore, multi-staged procedures of isolation and purification, low efficiency of obtained product, and methodological variations are evaluated as fundamental parameters that limit the therapeutic projection of PELNs in nano medicine and the pharmaceutical sector.

The literature is limited about how PELNs form, carry contents, and function in cells. This situation prevents the usage of vesicles in a safe and controlled manner [72]. One of the biggest challenges of PELN technology is preserving bioactivity after isolation and standardizing storage conditions. Despite their present potential at industrial scale, structural variability of methodological differences is the biggest obstacle in a safe drug distribution system. In this context, determination of source-based and methodological standards that have global validity is the common step that accelerates the transition process of PELNs from laboratory to clinics [97].

Large-scale and multi-diverse omics analyses play a key role in the strategies of precision medicine of these nanoparticles that are directed specifically at human pathologies and targeted treatment via decoding unique biomolecular signatures of different PELN classes [98]. Reliability of PELN-based treatments should be supported with broad-spectrum in vitro and in vivo models. Even if existing omic technologies are promising when presenting a biomolecular profile, the lack of standard biomarkers is a common technological gap that sidetracks quality control and data interpretation of these vesicles [96,97]. Thus, bioinformatic pipelines native to these data sets and specialized calculating algorithm designs are becoming a requirement [96]. The commercialization process of PELN technology should be built on two common columns: clinical verification and regulatory clarity. Even if obtained safety data from in vivo models proves the therapeutic value of this technology, the dilemma of ‘food contribution’ and ‘new generation food’ of products slows down the industrial-scale standardization. In this context, a thorough evaluation of the legal positioning of PELNs is required [96].

As mentioned in Section 4, the prebiotic potential of PELNs is not independent from biological and environmental variabilities in the host. The determining role of factors such as microbiota diversity, age, and diet on PELN response emphasizes again that the model of ‘one prescription that suits everyone’ is insufficient. Thus the customizable structure that PELNs show seizes a critical opportunity for personalized treatment protocols that target reshaping microbiota individually.

## 7. Future Perspectives

The biological effects of PELNs are dependent on distribution native to the kind. This unique composition produces functional outcomes that differ for every molecule class in the gut microbiota [25,74]. It has been found that PELNs differently regulate microbial balance and metabolical processes based on their sources. In this context, some PELN species trigger the production of bacteria that produce SCFA, whereas the other ones focus on protecting the mucosal barrier integrity and homeostasis [73,75]. Selective effects of PELNs on the gut microbiota are mediated through the miRNAs and lipid structures they contain. miRNAs that internalize into cells constitute a targeted biological response via manipulating the bacterial gene expression and metabolical processes whereas lipid composition acts by “addressing” which groups of bacteria will take up these particles [23,35]. PELNs derived from various fruits and vegetables have the ability to directly reach the guts via passing through gastric fluid. They take on critical roles such as reducing inflammation, repairing epithelial and balancing microbiota [16,80]. The findings prove that PELNs separate from classical prebiotics that enhance only the general population of microorganisms. PELNs become prominent as a complex regulator that can directly modulate targeted microbial functions and also immunometabolic pathways of the host with the help of biological signals that they contain [75]. Approach of “precision prebiotic exosomes” initiates a new-generation prebiotic engineering paradigm that uses a complex relationship between the plant source and microbial response as a base. This strategy foresees the usage of PELNs that are customized from native to individual microbiota profiles and expected therapeutic results. Being natural, bioavailable and applicable orally makes PELNs a transformational tool for functional food innovations and also targeted microbiota modulation [16,75]. PELNs, in opposition to the animal-derived exosomes, have some critical advantages, such as ethical suitability, sustainability, and production scalability. Their successful isolation from common agricultural products makes their integration into the existing food supply chain easier. With their low immunogenicity and being orally applicable, PELNs are an ideal candidate for new-generation prebiotic platforms that are sustainable at an industrial scale and are eco-friendly. PELNs can effectively control microbial processes in the gut when combined with dietary fibers and postbiotics. The fermentation ability of fibers and the biological signaling ability of PELNs form new-generation synbiotic platforms that regulate microbiota spot-on via coming together. This strategic cooperation allows more effective and science-based functional foods to be produced. Integration of PELNs into food matrices prepares an innovative ground to increase bioactivity of functional foods [99]. For instance, integration of blueberry-derived PELNs into beverages or capsulation of curcumin-loaded PELNs with polysaccharide matrices can optimize the stability of the active compounds and their bioavailability. However, the literature remarks that the functionality of these nanoparticles is tightly bound to the membrane integrity. Notably, high temperature and extreme pH values can cause structural disruptions. Thus, stabilization strategies such as spray-drying, microcapsulation or the use of preservative matrices would fill a critical research gap [71].

## 8. Conclusions

The understanding of traditional prebiotics classified these compounds as indigestible dietary elements that only the promote growth of the beneficial microorganisms [100]. However, this limited definition is not enough to include the critical role in targeted transmitting, structural complexity, and host-microbiota interactions of bioactive molecular signals. At this point, PELNs are included in the literature as a new generation of prebiotic carriers that combine nanoscale advantages with biological content. These structures that can carry sensitive charges by protecting them, such as lipids, proteins, and RNAs, are converted from the term prebiotic into functional and interactive nanosystems, separating them from a basic food source term.

Preclinical and clinical data that are outlined in Table 2 verify the critical role of PELNs in the regulation of gut homeostasis. The effects of these particles on microbiota are not limited to only the changes at the population level but also to the support of vital functional achievements such as SCFA production increasing and epithelial barrier strengthening. These findings present the potential of PELNs’ to be a strong therapeutic agent in inflammatory phases and infection-based dysbiosis situations, while mentioning that comprehensive translational studies are required to transfer this potential into clinical applications. Incorporation of PELNs into functional food systems shows that biotechnology, nutrition sciences, and microbiota studies converge on a strategic point. These structures that show outstanding biocompatibility can become prominent as a critical instrument in the development of sensitive personal nutrition models. Microbiota profiling studies supported by multi-omics technologies and data-driven analysis can facilitate the determination of functional foods that are suitable for unique microbial maps of individuals. Within this period, PELNs can be located as customizable bioactive carriers that transform traditional understanding of supplements and allow personalized diet programs. However, it is required to make progress in standardization, legal classification, and comprehensive clinical verifications in transforming this potential into a commercial and clinical reality.

## Figures and Tables

**Table 1:** Comparative overview of major isolation techniques for PELNs.

Method	Yield	Purity	Processing Time	Advantages	Limitations	References
Ultracentrifugation	High	Moderate	Long	Widely used standard method; scalable.	Co-isolation of protein aggregates and debris; requires specialized equipment.	[67,68]
PEG 6000	High	Low	Short	Simple, cost-effective, suitable for large volumes.	High risk of co-precipitating contaminants.	[70]
SEC	Moderate	High	Moderate	High purity; preserves vesicle integrity.	Lower yield; requires column setup and optimization.	[69,70]

**Table 2:** Clinical and Preclinical Applications of Plant-Derived Exosomes.

Clinical or Preclinical Applications	Population	Study Design	Dose	Duration	Results	References
Anti-inflammatory and barrier protection	Caco-2 human intestinal epithelial cell line	Caco-2 intestinal epithelial cells stimulated with lipopolysaccharide (LPS); barrier functions and inflammatory markers assessed by application of garlic-derived ELNs.	1, 5, 10 µg/mL garlic-derived ELNs	24 h	Barrier strengthened, tight-junction proteins increased, inflammatory cytokines decreased.	[85]
Colitis attenuation and barrier restoration	Male C57BL/6J mice	Colitis induced with dextran sodium sulfate (DSS); garlic-derived ELNs orally administered; clinical and molecular outcomes evaluated.	20, 100, 500 mg/kg (oral)	15 days	Colitis severity decreased, barrier proteins increased, inflammatory cytokines reduced; 100 mg/kg was most effective.	[85]
Colitis treatment via tissue repair and barrier strengthening	DSS colitis mice	Oral GDEN; intestinal stem cell activation and tissue regeneration assessed.	Not specified.	Not specified.	The barrier strengthened, repair increased, inflammation reduced.	[80]
Barrier improvement and inflammation control	DSS colitis mice	Oral GELN; epithelial and immune cell uptake examined.	Not specified.	Not specified.	Barrier integrity improved, inflammation suppressed.	[80]
Immune modulation to alleviate colitis	DSS and Rag1−/− mice	Sulforaphane-loaded broccoli-derived PELN; DC modulation evaluated.	Not specified.	Not specified.	Immune balance improved, inflammation markedly reduced.	[80]
Prevention and alleviation of colon disease	DSS colitis mice	Oral tea leaf-derived PELN; antioxidant and immune effects analyzed.	Not specified.	Not specified.	Oxidative stress reduced, barrier strengthened, inflammation lowered.	[80]
Clinical translation potential in humans	Refractory IBD patients	Safety and efficacy; GELN ± curcumin compared	Not specified.	Not specified.	Safety and anti-inflammatory effects under evaluation (results pending)	[80]
Antioxidant and anti-aging skin effects	Human dermal fibroblasts	In vitro cellular uptake and functional assays	PELN mix from grape, blood orange, tangerine, papaya, pomegranate	Up to 72 h	Efficient cellular uptake, antioxidant defense enhancement, improved mitochondrial homeostasis, anti-aging associated responses.	[86]
Barrier repair and tissue regeneration	Human dermal fibroblasts (monolayer)	In vitro scratch (wound-healing) assay	PELN mix	Until wound closure	Accelerated wound repair, increased collagen I, MMP-9 and vimentin expression at wound site	[86]
Cancer cell viability assessment	Human triple-negative breast cancer cell line (MDA-MB-231)	Cancer cells were treated with different GDEN concentrations and viability was measured using the MTT assay.	Increasing concentrations of GDENs	24 h	Cell viability decreased significantly in a dose-dependent manner.	[42]
Oral mucositis prevention in chemoradiation	Head and neck cancer patients (*n* = 60) under chemoradiation	Randomized, open-label trial (GDENs vs. standard care for oral mucositis)	GDEN-rich powder (oral) daily	35 days (concurrent with radiation therapy)	GDEN significantly reduced oral mucositis severity during chemoradiation without causing notable toxicity (Phase I).	[87]
Colitis and Colitis-Associated Cancer	Mouse colitis and colon cancer (CAC) models	Oral prophylactic and therapeutic treatment in acute and chronic colitis	~2 mg/day of GELNs	~10–14 days (prevention and treatment phases)	GELNs reduced colitis severity and tumor burden by enhancing intestinal barrier repair, suppressing inflammation, and limiting oxidative stress.	[31]
Colitis (Anti-inflammatory)	Mice with DSS-induced colitis	Oral Broccoli-derived ELN treatment, prevention and therapy in IBD model	250 µg/mouse per dose	~10 days pre-DSS + 7–12 days during colitis	Broccoli-derived ELNs delivered sulforaphane to activate AMPK in DCs, inducing tolerogenic immune responses and reducing colitis severity, weight loss, disease activity, and histological damage.	[88]
DSS Colitis	Mice with DSS-induced colitis	Oral grapefruit-derived ELN therapy during IBD	Not explicitly reported(low-dose grapefruit-derived ELNs, oral gavage)	~7–10 days (acute colitis phase)	Grapefruit ELNs alleviated colitis by macrophage-mediated anti-inflammatory modulation.	[89]
Colitis (Anti-inflammatory and antioxidant)	Mice with DSS-induced IBD (plus in vitro macrophage assays)	Two-dose oral treatment (low vs. high dose) in IBD model, with mechanistic analyses	5 mg/mL and 10 mg/mL Ginseng-derived ELNs (≈1–2 mg/mL mouse, oral)	3 days pretreatment + 7 days DSS (10 days total)	Ginseng ELNs reduce colitis through anti-inflammatory, antioxidant, barrier-protective, and microbiota-modulating effects.	[90]
Oral Cancer (Anti-tumor and anti-inflammatory)	Human oral squamous carcinoma (OSCC): cell lines and xenograft mice	In vitro cytotoxicity and in vivo efficacy test, alone and combined with chemotherapy (5-FU)	Bitter melon-derived ELNs ~100 µg/mL in vitro; in vivo ~10 µg/g via i.p. (with 5-FU 20 mg/kg)	~4 weeks (tumor growth experiment)	Bitter melon ELNs suppressed OSCC growth and enhanced 5-FU efficacy by inhibiting NLRP3-mediated chemoresistance.	[91]

## Data Availability

No new data were created or analyzed in this study. Data sharing is not applicable to this article.
